# Optimizing Outcomes in Oncoplastic Breast-Conserving Surgery

**DOI:** 10.3390/jcm14134806

**Published:** 2025-07-07

**Authors:** Aileen Gozali, Merisa Piper

**Affiliations:** 1 School of Medicine, University of California San Francisco, S-245, 513 Parnassus Ave. Suite, San Francisco, CA 94143, USA; aileen.gozali@ucsf.edu; 2 Division of Plastic and Reconstructive Surgery, Department of Surgery, University of California San Francisco, 3rd Floor, 1825 Fourth Street, San Francisco, CA 94158, USA

**Keywords:** oncoplastic breast-conserving surgery, oncoplastic surgery, volume displacement and replacement techniques, surgical optimization in breast cancer

## Abstract

Oncoplastic breast-conserving surgery (OBCS), or oncoplastic surgery, has revolutionized the surgical management of breast cancer by integrating oncologic principles with reconstructive techniques to optimize both cancer control and aesthetic outcomes following breast-conserving surgery (BCS). Since its inception in the 1980s, the field has evolved significantly, incorporating a range of volume displacement and volume replacement strategies to restore breast contour after partial mastectomy. This review explores the current practices and key surgical considerations of OBCS. It highlights the role of preoperative multidisciplinary planning, patient selection, anatomical and vascular knowledge, and intraoperative technique in optimizing results. Barriers to access—including disparities in training, insurance, and geographic availability—are addressed, alongside efforts by professional societies like the American Society of Breast Surgeons (ASBS) to standardize definitions and practices. The review also outlines strategies for minimizing complications and enhancing oncologic, reconstructive, and patient-reported outcomes. By offering a comprehensive framework for clinical decision-making, this paper aims to support broader adoption and refinement of OBCS as a standard component of breast cancer care.

## 1. Introduction

Oncoplastic breast-conserving surgery (OBCS), or oncoplastic surgery, integrates oncologic and plastic surgery principles to optimize both cancer control and aesthetic outcomes following breast-conserving surgery (BCS). It emerged when breast-conserving therapy (BCT)—lumpectomy or partial mastectomy followed by radiotherapy—proved equally effective as mastectomy for early-stage breast cancer [1,2]. However, BCT was often associated with unsatisfactory aesthetic outcomes, such as contour deformities, fibrosis, and scarring, especially when more than 10% of breast tissue was excised [3,4]. Oncoplastic techniques were introduced to address these challenges and have since shown lower re-excision and positive margin rates [5,6], similar if not fewer complications [7,8,9,10], and enhanced patient-reported outcomes compared to BCS alone [11]. Today, OBCS is accepted as the standard of care for patients undergoing partial mastectomy worldwide [12,13]. Since its introduction in the 1980s by Dr. Werner Audretsch [14], the field has evolved continuously, emphasizing optimization of oncologic, functional, aesthetic, and patient-reported outcomes. This review summarizes the evolution, techniques, and classifications of OBCS, and outlines strategies for optimizing outcomes.

## 2. Evolution of OBCS and Barriers

Surgical management of breast cancer has evolved significantly, transitioning from the radical Halstedian mastectomy in the late 19th century to the widespread acceptance of BCT by the 1990s, driven by advances in surgical techniques, pathologic evaluation, chemotherapy, and radiotherapy [1,2,15,16,17,18]. In 1994, Dr. Werner Audretsch introduced the concept of “oncoplastic surgery”, integrating principles of oncologic and reconstructive surgeries [14]. Early oncoplastic techniques adapted traditional breast reduction and mastopexy methods—termed “volume displacement” approaches—to repair partial mastectomy defects [19,20]. By the early 2000s, volume replacement techniques such as locoregional flaps expanded the ability to restore breast shape after larger resections [21,22,23,24].

Despite the evolution of techniques, access to OBCS remains limited [25]. A major barrier is the lack of standardized training among breast surgeons, resulting in variability in expertise across practice settings [26,27,28]. Models of delivery of OBCS also differ internationally. In the United Kingdom, a single-surgeon model predominates, with breast surgeons who received training in oncoplastic techniques performing both the oncologic resection and reconstructive procedures [29]. In contrast, a two-surgeon model is more common in the United States and Australia, where a breast surgeon performs the oncologic resection and a plastic surgeon performs the immediate reconstruction [30]. Complex volume replacement procedures, in particular, require coordination with experienced plastic surgeons often found in high-volume academic centers. Additional barriers include insurance limitations, geographic disparities, referral biases, and racial inequities [31,32,33]. Historically, the absence of a consistent definition of OBCS created confusion not only among providers and trainees but also among patients seeking breast cancer treatment. Furthermore, the lack of specific billing codes for OBCS has further impeded broader adoption [28,34].

**Figure 1:**
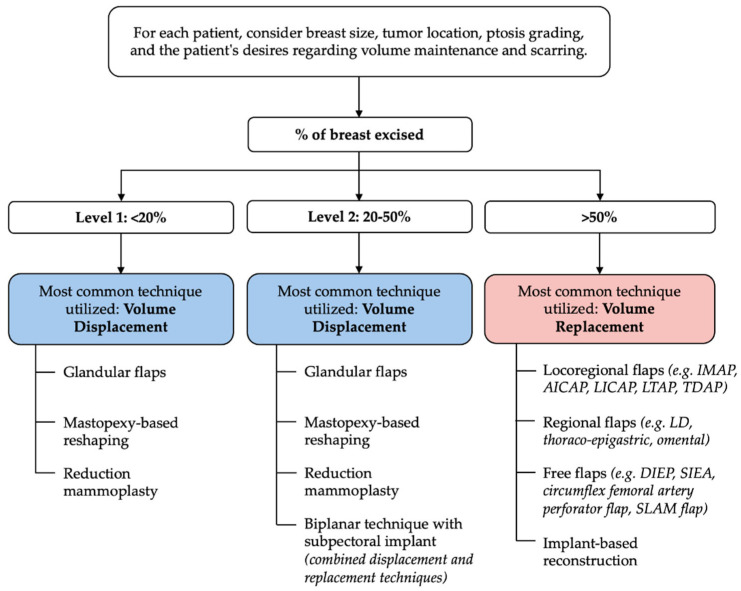
Suggested guide to common oncoplastic techniques based on the American Society of Breast Surgeons (ASBS) classification system. This schematic categorizes oncoplastic approaches according to the percentage of breast volume excised: Level 1 (<20%), Level 2 (20–50%), and Volume Replacement (>50%). These thresholds serve as general guidelines rather than strict cut-offs. Surgical decision-making should be individualized, taking into account factors such as breast size, tumor location, ptosis, availability of remote tissue, and the patient’s preferences regarding volume preservation and scarring.

To address these challenges, the American Society of Breast Surgeons (ASBS) developed a consensus definition and classification system to standardize practice and expand access [35]. The ASBS defines OBCS as “breast conservation surgery incorporating an oncologic partial mastectomy with ipsilateral defect repair using volume displacement or volume replacement techniques, with contralateral symmetry surgery as appropriate”. Building upon previous frameworks such as Clough’s excision volume and surgical complexity-based model focusing primarily on volume displacement [36], the ASBS definition classifies oncoplastic procedures into volume displacement (Level 1: <20% excision; Level 2: 20–50% excision) and volume replacement (>50% excision) categories, explicitly including tissue recruitment techniques. The 20% and 50% thresholds serve as guidelines rather than rigid cut-offs, allowing for individualized decision-making based on patient anatomy and goals. A suggested guide to common oncoplastic techniques based on the ASBS classification system can be found in Figure 1. Notably, while the ASBS definition was designed primarily for clinical clarity rather than billing purposes, it remains compatible with Current Procedural Terminology (CPT) coding systems, thus supporting broader integration of OBCS into routine breast cancer care [35].

Efforts to expand access to OBCS have also focused on strengthening training and credentialing pathways. In the United States, the ASBS offers formal oncoplastic surgery certification program to recognize surgeons meeting benchmarks in clinical experience, procedural training, and quality assurance in OBCS [37]. In the United Kingdom, growing patient demand for immediate reconstruction following BCS led to the development of a national breast surgery curriculum mandating oncoplastic training, hands-on simulation courses, and the emergence of “stem breast surgeons”—a surgeon trained in either breast or plastic surgery who receives cross-specialty education in both oncologic resection and reconstruction, enabled by a joint initiative between the Association of Breast Surgery (ABS) and the British Association of Plastic, Reconstructive and Aesthetic Surgeons (BAPRAS) [38]. More recently, new initiatives such as an online Master of Surgery degree in Oncoplastic Breast Surgery based at the University of East Anglia in the UK [39], and changes to the surgical curricula that may enable breast surgeons to specialize in more advanced reconstructive skills such as autologous flap reconstruction without general surgery on-call duties, have further expanded training pathways. Regardless of the model, it is essential that practices include providers—either individually trained or through collaborative teams—capable of delivering OBCS as a standard offering. This necessitates not only theoretical training and practical skill development but also formal oversight, documentation, and credentialing to ensure surgical quality. Greater transparency in surgeon qualifications also empowers patients to identify and advocate for OBCS as a treatment option.

## 3. Pre-Operative Evaluation

Patients with breast cancer undergoing lumpectomy or partial mastectomy should be referred for evaluation and education regarding OBCS. A multidisciplinary approach involving surgical oncology, medical oncology, radiation oncology, and plastic surgery has been shown to improve outcomes [40]. Preoperative assessment must systematically evaluate factors that influence both oncologic and reconstructive planning, including tumor size, focality, estimated resection size relative to breast volume, tumor location, degree of ptosis, skin quality, history of prior radiation, and the anticipated need for adjuvant therapies.

The most critical oncologic principle remains complete tumor excision with negative margins. OBCS often allows larger resections without compromising aesthetic outcomes, reducing positive margin rates compared to standard partial mastectomy [41]. For tumors initially too large for breast conservation, neoadjuvant therapy can be employed to downstage the disease [42,43,44]. In these cases, pre- and post-treatment breast MRI is recommended to assess response and reevaluate surgical candidacy [45,46]. Patients’ goals and preferences are equally vital; factors such as desired breast size, tolerance for incisions, and acceptance of surgical complexity must be discussed thoroughly. Setting realistic expectations regarding symmetry, breast contour, potential radiation effects, and possible complications is essential for patient satisfaction and informed consent.

Detailed anatomical understanding is crucial for surgical planning. Successful OBCS relies on maintaining perfusion to glandular flaps and preserving nerve supply to optimize nipple-areolar complex (NAC) viability and minimize fat necrosis. Pedicle design must account for vascular anatomy. Tumor location, body habitus, and breast size guide technique selection: patients with larger, ptotic breasts are better suited for volume displacement techniques, whereas those with smaller breasts may require volume replacement strategies using flaps. Aesthetically placed incisions (e.g., periareolar, inframammary, or axillary) and meticulous glandular rearrangement are essential to optimize outcomes and minimize visible scarring. Preoperative planning also considers the need for contralateral symmetry procedures, which may be performed synchronously or delayed based on patient factors. Notably, breast reduction itself may confer a reduced breast cancer risk in older women, adding an additional benefit to symmetry-achieving surgery [47,48].

## 4. Classifications of Oncoplastic Techniques

### 4.1. Volume Displacement Techniques

Volume displacement techniques are the most commonly employed form of oncoplastic techniques, particularly in patients with moderate-sized breasts, small-to-medium tumors, and mild ptosis (Grade I) [49]. These approaches involve rearrangement of the residual breast tissue after tumor excision to restore breast contour, typically resulting in a modest reduction in breast volume. Displacement strategies are most appropriate when up to 20–50% of breast volume is excised [35], and patients must be counseled about the expected decrease in breast size. Proper patient selection is key; women with adequate glandular tissue, some skin laxity, and moderate breast size achieve the best aesthetic outcomes, as these factors support tissue mobility.

**Figure 2:**
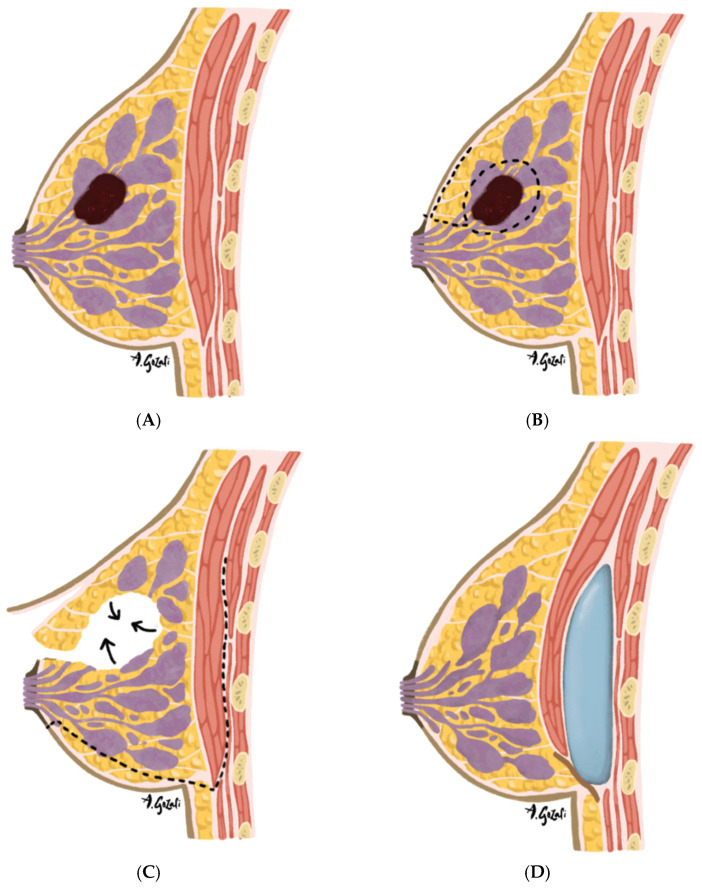
(**A**–**D**). Biplanar oncoplastic surgery is a hybrid technique combining volume displacement and volume replacement strategies in patients with small, non-ptotic breasts undergoing breast-conserving surgery. This method is indicated when tumor resection would result in significant contour deformity and volume reduction that cannot be corrected with glandular rearrangement alone. A prosthetic device is placed in a subpectoral pocket to restore volume while minimizing additional donor site morbidity. The technique is especially suitable for peripheral tumors in the upper or outer quadrants. (**A**) Preoperative tumor localization: Tumor located in the upper outer quadrant of the breast—a favorable site for biplanar oncoplastic surgery due to ease of access and preservation of vascularity during rearrangement; (**B**) Tumor Excision: Incision design is based on tumor location, with common approaches including circumvertical and periareolar patterns. Skin flaps are elevated within the plane of the investing fascia, and the tumor is excised with a limited margin of surrounding healthy breast tissue. The specimen is oriented, weighed, and submitted for pathological analysis; (**C**) Parenchymal rearrangement following negative margin confirmation: Tissue rearrangement is performed through parenchymal and skin flap mobilization to fill the resection cavity and maintain breast contour. Attention is given to preserving vascular supply to minimize the risk of necrosis; (**D**) Subpectoral implant placement: A subpectoral pocket is developed by elevating the pectoralis major muscle from its inferior-lateral border via a transparenchymal tunnel or secondary inframammary fold incision. A small prosthetic device (implant or tissue expander) is inserted to replace lost volume and restore symmetry.

A variety of glandular rearrangement techniques are employed depending on tumor location and the volume of resection [36]. These include glandular flap advancement [36], rotational glandular flaps [50,51], mastopexy-based reshaping [52,53,54], and reduction mammoplasty techniques [36,55]. Familiar skin incision designs, such as vertical or Wise-pattern incisions, are adapted to accommodate oncologic resection patterns [52,56]. The choice of pedicle and skin excision pattern is influenced by the tumor’s quadrant, breast size, and degree of ptosis. Novel strategies, such as the biplanar oncoplastic approach—which combines glandular reshaping with small-volume subpectoral implants—are being explored in selected patients to help restore volume symmetry (Figure 2) [57]. This is an example of emerging techniques that integrate volume replacement elements into displacement frameworks to address limitations of volume displacement approaches.

An intimate knowledge of breast anatomy—including vascular, lymphatic, and neural structures—is fundamental to the success of volume displacement procedures [58]. The breast is supplied by an anastomotic arterial network derived from the internal mammary, axillary, and intercostal arteries, which also forms the vascular basis for most glandular flaps used in OBCS. This is complemented by a robust subdermal venous plexus through which venous drainage occurs. Equally important is the preservation of lymphatic drainage and sensory innervation, particularly to the NAC, which receives input primarily from the fourth intercostal nerve and branches of the medial intercostal and supraclavicular nerves. Respecting these anatomical territories during dissection helps reduce complications such as flap necrosis, venous congestion, and postoperative sensory deficits such as postoperative numbness and neuropathic pain [59].

Displacement techniques are not without limitations. Extensive resections and undermining of tissue may compromise flap mobility or blood supply, increasing the risk of complications such as fat necrosis, wound dehiscence, delayed healing, or even partial or complete NAC loss [60].

### 4.2. Volume Replacement Techniques

Volume replacement techniques utilize autologous tissue to restore breast shape and volume after partial mastectomy, particularly in patients with small-to-moderate-sized breasts, high tumor-to-breast size ratios, or insufficient residual breast tissue following resection [61]. These approaches are most beneficial when more than 50% of the breast volume is removed, or in cases of 20–50% volume loss when the patient desires preservation of breast size [35]. Flap choice is determined by tumor location, the volume required for reconstruction to achieve the desired breast size, and patient-specific factors such as tissue availability and vascular anatomy.

**Figure 3:**
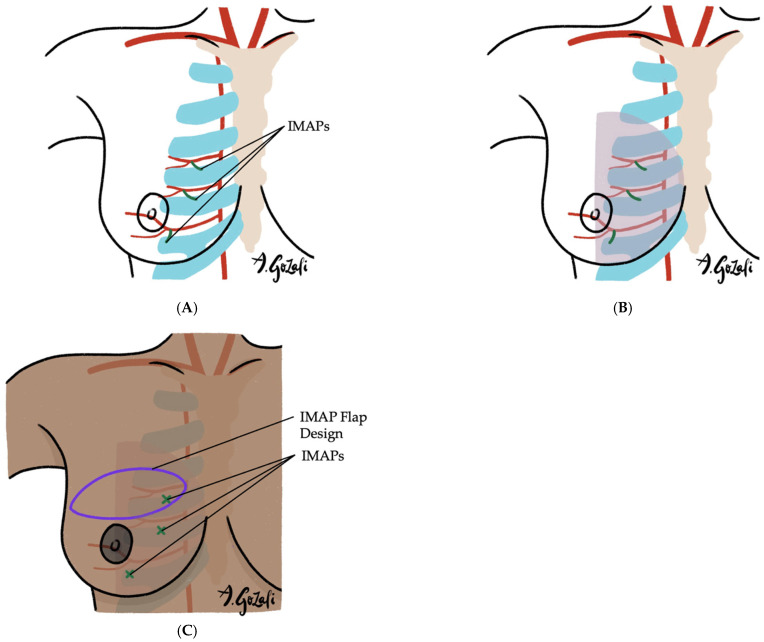
(**A**–**C**). Internal Mammary Artery Perforator (IMAP) Flaps. (**A**) Vascular supply of IMAP flaps is based on the perforating branches of the internal mammary artery; (**B**) IMAP flaps are especially suitable for defects in the medial half of the breast; (**C**) IMAP flap designs are versatile and can be tailored according to the location of the defect.

**Figure 4:**
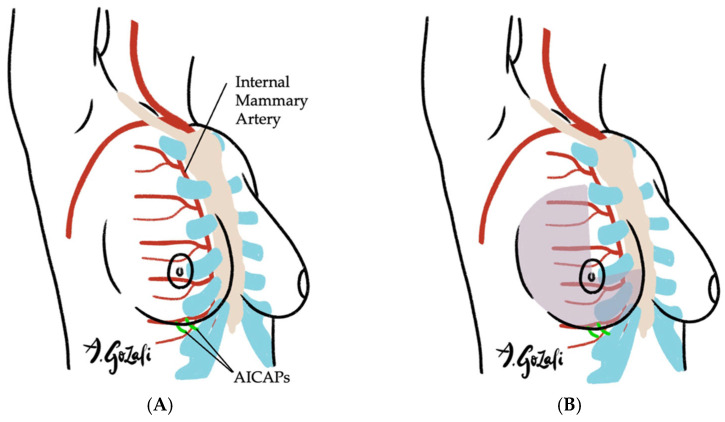

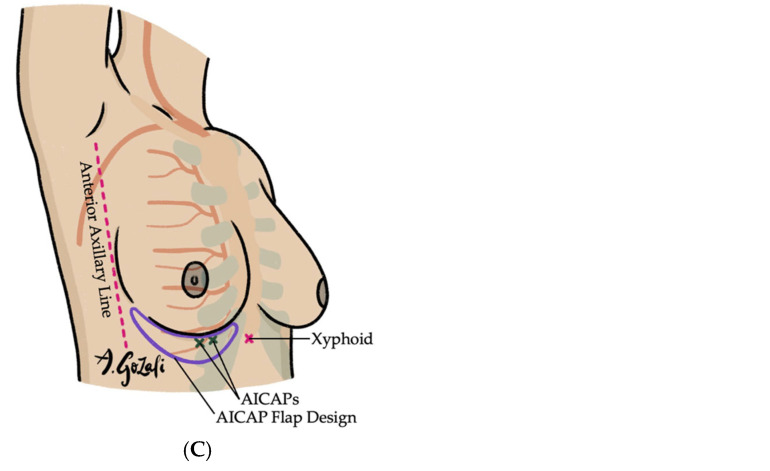
(**A**–**C**). Anterior Intercostal Artery Perforator (AICAP) Flaps. (**A**) Vascular supply of AICAP flaps is based on the perforating branches of the anterior intercostal artery perforator, which branches off the internal mammary artery; (**B**) AICAP flaps are especially suitable for defects located in lateral breast or inferior medial quadrant; (**C**) When designing AICAP flaps, the upper boundary is typically the IMF, with the flap width spanning from the anterior axillary line to the xyphoid, subdivided into thirds and the appropriate third is chosen based on location of the defect and the perforators.

**Figure 5:**
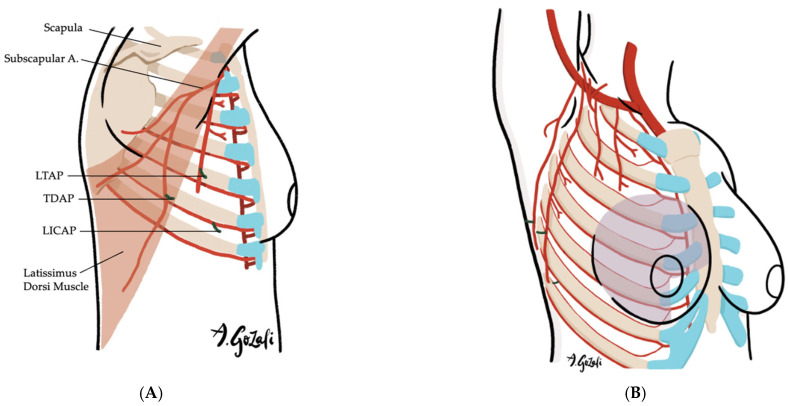

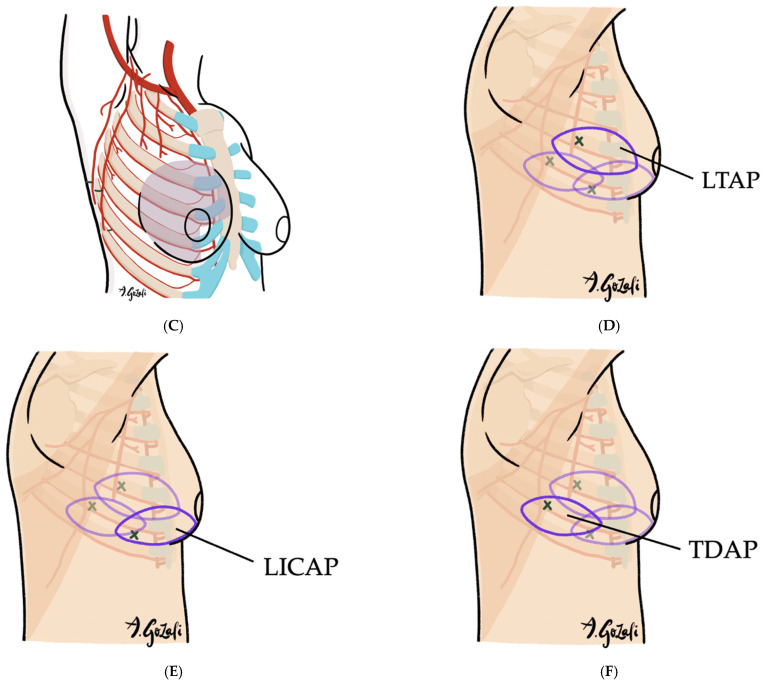
(**A**–**F**). Lateral Thoracic Artery Perforator (LTAP) Flaps, Thoracodorsal Artery Perforator (TDAP) Flaps, and Lateral Intercostal Artery Perforator (LICAP) Flaps. (**A**) Vascular supply of LTAP flaps is based on perforators of the lateral thoracic artery (a branch of the axillary artery); LICAP flaps are supplied by perforators of the lateral intercostal artery (a branch of the posterior intercostal artery); TDAP flaps are supplied by the perforators of the thoracodorsal artery (a branch of the subscapular artery); (**B**) LTAP and LICAP flaps are suitable for lateral breast defects; (**C**) TDAP flaps are suitable for large surface area defects located in lateral breast or superior medial quadrant; (**D**–**F**) The flaps are designed on the lateral chest wall based on the location of the perforators.

**Figure 6:**
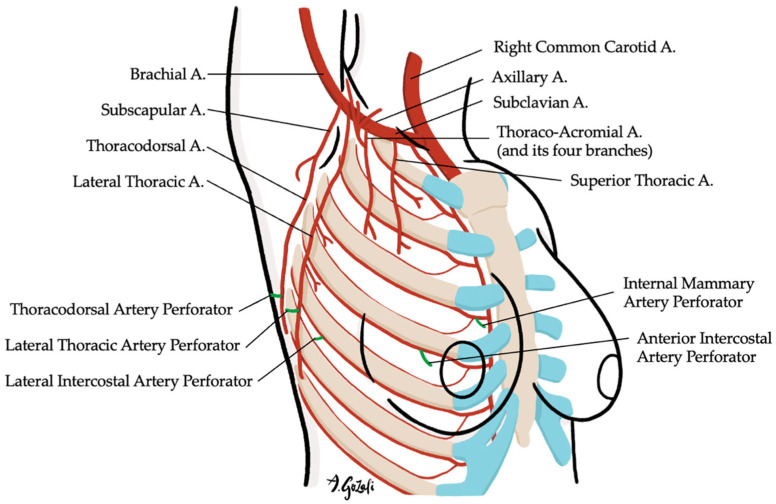
Chest wall perforators available for locoregional flap as volume replacement techniques.

Locoregional flaps based on chest wall perforators offer reliable options for local tissue recruitment with minimal donor site morbidity and no need for microvascular anastomosis. These include the internal mammary artery perforator (IMAP) flap [62] (Figure 3), anterior intercostal artery perforator (AICAP) flap [63] (Figure 4), lateral intercostal artery perforator (LICAP) flap [64,65], lateral thoracic artery perforator (LTAP) flap [65], and thoracodorsal artery perforator (TDAP) flap [66,67,68,69] (Figure 5). Ordered anatomically from medial to lateral, these flaps differ in arc of rotation and tissue availability (Figure 6). Although comparative volumetric data are limited, reports suggest the TDAP flap can address defects involving more than 20% of breast volume [70], while the LICAP and IMAP flaps can replace up to 100 g and 125 g of tissue, respectively [62,71]. However, these locoregional flaps may not always provide sufficient volume for larger defects, and they may result in additional chest wall incisions.

**Figure 7:**
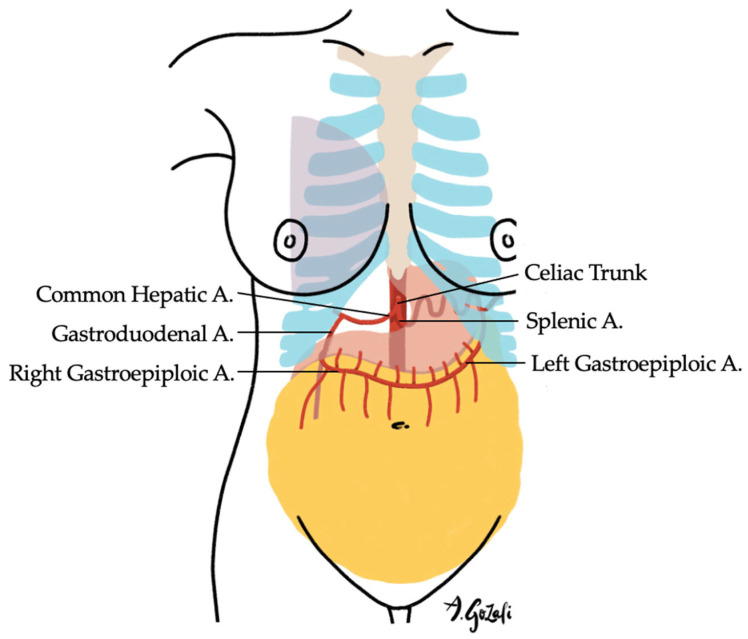
The Omental Flap is supplied by the left or right gastroepiploic arteries (one is usually more dominant than the other). It is suitable for medial breast defects, especially when >20% of breast tissue is excised.

Regional flaps such as the traditional latissimus dorsi (LD) flap, thoracoepigastric flap, and omental flap offer more substantial tissue volume without the complexity of microsurgical anastomosis. The LD flap remains a workhorse for volume replacement, particularly when chest wall perforators are insufficient [72,73]. The thoracoepigastric flap, supplied by perforators from the superior epigastric artery, is well-suited for inferior defects, both lateral and medial [61,74,75]. It is best used in patients with adequate subcutaneous tissue and skin in the upper abdomen, with no prior scarring in the ipsilateral upper abdomen, which may compromise the pedicle. The omental flap—harvested laparoscopically or via an open approach—offers pliability and robust vascularity, especially for medial or lower-pole reconstructions [76,77] (Figure 7).

**Figure 8:**
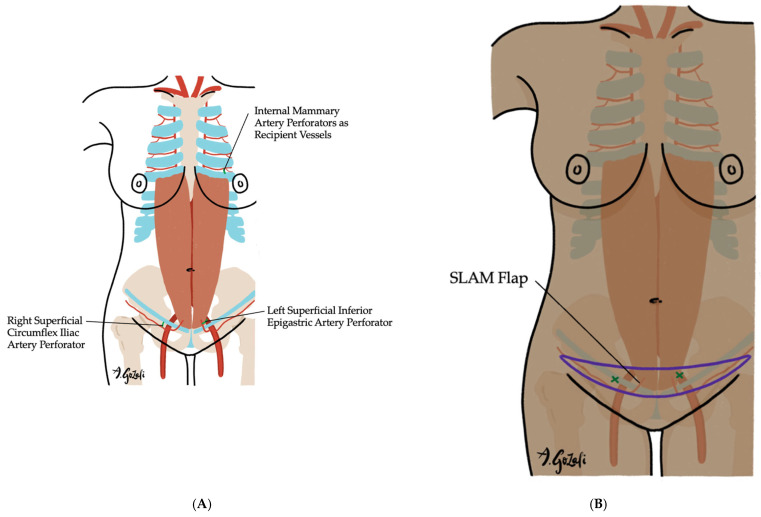
The Superficially based low-abdominal mini (SLAM) flap. (**A**) Vascular supply of the SLAM flap is determined by the dominant vessel perfusing the designed flap, typically either the superficial inferior epigastric artery or the superficial circumflex iliac artery. This flap is ideal for small-breasted, thin patients undergoing 20–50% volume excision who wish to preserve breast volume; (**B**) The flap is named for its flap design, which is a narrow strip of lower abdominal tissue that allows for preservation of abdominal donor site for future reconstruction need.

Free tissue transfers are reserved for extensive volume deficits or when previous surgeries, radiation, or chest wall scarring preclude the use of locoregional or regional options. Abdominally-based flaps such as the deep inferior epigastric perforator (DIEP) and superficial inferior epigastric artery (SIEA) flaps are most commonly used [78,79]. Smaller-volume free flaps, such as those based on the medial circumflex femoral artery perforator, may be useful particularly in central breast defects [80], and the superficially-based low-abdominal mini (SLAM) flap has recently been described as a means of providing modest volume replacement in thin patients while preserving future abdominal donor sites [81] (Figure 8). Free flap reconstruction remains technically demanding, with challenges including recipient vessel access and flap inset, especially in previously irradiated or surgically altered fields.

Complications in volume replacement include flap loss, donor site morbidity, and delayed wound healing. Success relies heavily on careful patient selection, preoperative imaging of perforator anatomy if indicated, and experienced surgeons. Despite the growing use of these techniques, there remains a lack of standardized reporting on key variables—such as preoperative breast size, tumor location, and positive margin definitions (ranging from 1–2 mm to <10 mm across institutions)—making comparison of outcomes difficult [7].

Table 1 summarizes the volume displacement and replacement techniques mentioned in this review as well as their indications (Table 1).

## 5. Outcome Optimization

Optimizing outcomes in OBCS requires a coordinated approach that integrates patient-reported, aesthetic, reconstructive, and oncologic goals. Among these, patient-reported outcomes have become increasingly central, as they reflect the patient’s own assessment of satisfaction, body image, and quality of life. Compared to standard BCS, OBCS consistently yields higher patient-reported outcome measures [11,82]. However, achieving high satisfaction depends on proper patient selection, technical execution, and managing expectations, particularly in the context of anticipated adjuvant therapy, which may alter the final cosmetic result.

Setting expectations before surgery is key to optimizing patient-reported outcomes. Patients should be informed not only about oncologic control but also about scar placement, potential loss of nipple or skin sensation, possible changes in breast shape due to adjuvant therapy, and the need for contralateral symmetrizing procedures. Whenever feasible, symmetry procedures should be performed during the same operation to enhance aesthetic outcomes, minimize recovery time, and reduce cost [83,84,85].

Success in OBCS also depends heavily on patient and procedural factors. Optimization of modifiable risk factors—such as body mass index (BMI), smoking cessation, and diabetes control—can reduce complication rates and improve healing. Unlike aesthetic breast surgery, OBCS involves tissue that has already been compromised by cancer excision, leading to reduced vascularity and altered architecture. Therefore, strategies that prioritize vascular preservation, minimization of tissue trauma, and avoidance of excessive tension during closure are essential for successful reconstruction.

From a reconstructive standpoint, the most common complications are early wound healing issues. Approximately 80% of complications after OBCS occur prior to initiation of radiation therapy, and about 8% of patients may experience delays in adjuvant treatment due to surgical complications [86]. Prevention of wound healing complications is therefore critical, as treatment delays can negatively impact oncologic outcomes. As tissue subjected to partial mastectomy already suffers disruption of vascular networks and structural integrity, key strategies include selecting robust pedicles based on vascular anatomy, minimizing closure tension, and handling tissue gently during glandular advancement to preserve vascular perfusion and promote wound healing. Strategies to minimize tension include selecting appropriate pedicles based on tumor location—such as using two shorter pedicles for lateral defects rather than one elongated pedicle [87]—to preserve perfusion and reduce complication rates. When advancing glandular tissue, sutures should be placed within sturdy glandular parenchyma rather than predominantly fatty tissue to avoid tearing, bleeding, and fat necrosis. Designing the resection cavity to collapse predictably, such as in the Benelli mastopexy where tissue is oriented to close radially around the NAC, helps maintain contour and NAC position [88]. Additionally, preserving tissue mobility by limiting dissection to either the subcutaneous plane or the chest wall fascia—but not both—reduces vascular disruption. Strategic incision planning (e.g., periareolar, inframammary, or crescent incisions) and careful tunneling techniques further support favorable cosmetic outcomes.

From an oncologic standpoint, the primary goal remains achieving negative margins while preserving the breast. OBCS facilitates wider excisions compared to standard partial mastectomy, reducing positive margin and re-excision rates [89]. However, the glandular rearrangement that defines OBCS can complicate intraoperative margin assessment. Techniques such as intraoperative margin assessment tool [90], ultrasound guidance, and frozen section analysis are invaluable tools for ensuring complete tumor removal. Still, definitions of an adequate margin vary across institutions as mentioned before, making multidisciplinary coordination and intraoperative judgement critical.

Proper undermining is essential for both oncologic access and effective glandular redistribution, but must be carefully balanced to avoid compromising vascularity. Skin and parenchymal undermining should be sufficient to allow for tension-free tissue advancement, especially for peripheral tumors. However, excessive undermining—particularly of the superficial fat layer—can increase the risk of fat necrosis and wound healing complications. Subareolar undermining should be minimized to preserve NAC vascularity and positioning. These technical details are critical to achieving both aesthetic and functional outcomes, particularly in patients undergoing adjuvant radiation.

Ultimately, successful OBCS hinges on integration: combining oncologic safety with aesthetic and functional goals, while maintaining alignment with patient expectations. A multidisciplinary, patient-centered approach—starting with counseling and extending through reconstruction and adjuvant therapy—is essential to maximize outcomes across all domains.

## 6. Conclusions

OBCS has transformed the landscape of surgical management of breast cancer by uniting oncologic safety with long-term aesthetic and functional restoration. The field has advanced from simple volume displacement procedures to a diverse spectrum of techniques—including complex volume replacement and free tissue transfer techniques—that now allow for tailored, patient-specific approaches. This evolution is accompanied by clear evidence of improved patient-reported outcomes and reduced rates of positive margins and re-excisions.

While technical progress has expanded the reconstructive toolbox, practical and systemic barriers—such as lack of access to trained providers and inconsistent reporting standards—continue to limit equitable implementation and comparative research. Recent efforts to address these challenges include the ASBS consensus definition and classification system, which provides a standardized framework for OBCS. Beyond improving clarity and accessibility, this system has shown potential as a predictor of clinical outcomes: a recent study demonstrated that patients undergoing Level II excision had significantly higher odds of developing delayed wound healing [91]. Importantly, growing evidence suggests that BCS with radiation therapy offers survival benefits compared to mastectomy in treating early-stage breast cancer, reinforcing the role of OBCS not just as a sound option but as the preferred treatment in eligible patients [92,93]. These findings underscore the need to further establish uniform outcome metrics, support prospective data collection, and ensure broader dissemination of OBCS expertise across diverse healthcare settings in order to expand patient access to oncoplastic techniques that improve both aesthetic and survival outcomes.

This review underscores the importance of optimizing outcomes across oncologic, reconstructive, aesthetic, and patient-reported domains. Whether through refined surgical strategies—such as minimizing closure tension, preserving pedicle perfusion, or tailoring flap choice to anatomy—or through preoperative planning and expectation setting, maximizing outcomes in OBCS requires an intentional, multidisciplinary, and patient-centered approach. Continued innovation, research, and policy support will be essential to ensure that the benefits of OBCS are equitably and consistently delivered to all patients undergoing BCS for breast cancer.

## Figures and Tables

**Table 1:** Summary of Oncoplastic Breast-Conserving Surgery Techniques and Their Indications.

Techniques		Indications
**Volume Displacement Techniques:** rearrangement of residual breast tissue after tumor excision to restore breast contour, require undermining the breast tissue from the skin and/or pectoral muscle. Typically results in modest volume reduction and patients should be counseled accordingly. Commonly employed in patients with moderate-sized breasts, small-to-medium tumors, and mild Grade I ptosis [49]. Density of glandular tissue (assessed using BIRADS score) would impact amount of undermining: lower density breasts have higher risk of fat necrosis after extensive undermining and thus should be limited [34]. Appropriate for when up to 20–50% of breast volume is excised [35].		
Glandular flap advancement		Vascular supply relies on advancement of adjacent glandular tissue into the cavity. Suitable for defects in almost all quadrants, though aesthetic outcomes are best for defects in upper outer quadrant. Main advantages include ease of mobilization and good aesthetic outcomes in lateral defects. Disadvantages include risk of deformity in the superior medial quadrant or the inferior half of the breast [36].
Rotational glandular flaps		Vascular supply is preserved through wide-based glandular tissue rotation into the excision cavity. Suitable for defects in the upper inner quadrant but applicable to all quadrants. Main advantages include better fill and contour in challenging locations such as the upper inner quadrant [50]. Disadvantages include limited use in lower-density breasts (a BIRADS score of 3–4 is ideal) or those with history of smoking or radiation. Novel variations include the dermoglandular rotation flap with subaxillary advancement [51].
Mastopexy		Vascular supply is dependent on pedicle design. Suitable for access to multiple quadrants, with different incision design allowing access to different quadrants of the breast [54]. For example, doughnut mastopexy (also known as Benelli or round-block mastopexy) allows access to all quadrants; crescent mastopexy allows access to the upper inner and outer quadrants; wise-pattern design is suitable for ptotic breasts and allows for wide exposure [50]; bat-wing excision pattern (standard keyhole incision with laterally-extended triangular incisions) is suitable for a central or upper inner quadrant defect [53]. Main advantages include simultaneous reshaping and access to tumor location.
Reduction mammoplasty		Vascular supply is dependent on pedicle choice, which is determined by tumor location [36,55]. For example, inferior pedicle mammoplasty is suitable for defects in the upper pole, while superior pedicle mammoplasty is suitable for defects in the lower pole. Main advantages include simultaneous volume reduction especially in patients with macromastia.
**Volume Replacement Techniques:** utilizes autologous tissue to restore breast shape and volume. Flap choice is determined by tumor location, the volume required for reconstruction to achieve the desired breast size, and patient-specific factors such as tissue availability and vascular anatomy. Commonly employed in patients with small-to-moderate-sized breasts, high tumor-to-breast size ratios, or insufficient residual breast tissue following resection [61]. Appropriate for when more than 50% of the breast volume is removed, or in cases of 20–50% volume loss when the patient desires preservation of breast size [35].		
Locoregional Flaps	Internal Mammary Artery Perforator (IMAP) flap	Vascular supply is through perforators of the internal mammary artery. Suitable for defects in medial half of the breast. Main advantages include reliable cosmetic outcomes and versatile flap design [62].
	Anterior Intercostal Artery Perforator (AICAP) flap	Vascular supply is through perforators of the anterior intercostal arteries. Suitable for defects in lateral breast or inferior medial quadrant. When designing the flap, the upper boundary is typically the IMF, with the flap width spanning from the anterior axillary line to the xyphoid, subdivided into thirds and the appropriate third is chosen based on location of the defect. Main advantages include well-concealed donor site [63].
	Lateral Intercostal Artery Perforator (LICAP) flap	Vascular supply is through perforators of the lateral intercostal artery. Suitable for defects located in the lateral breast, especially in post-weight-loss patients with excess lateral chest wall skin and adipose tissue. Main advantages include ease of dissection and sparing of the latissimus dorsi muscle. Disadvantages include a short pedicle length, limiting its use to lateral defects only [64,65].
	Lateral Thoracic Artery Perforator (LTAP) flap	Vascular supply is through perforators of the lateral thoracic artery. Suitable for lateral breast defects, often used in combination with LICAP flaps. Main advantages include minimal donor morbidity compared to the TDAP flap and sparing of the latissimus dorsi muscle; it can be fully or partially mobilized compared to the LICAP flaps. Disadvantages are similar to other lateral flaps, with limited reach [65].
	Thoracodorsal Artery Perforator (TDAP) flap	Vascular supply is through perforators of the thoracodorsal artery, a branch of the subscapular artery. Suitable for large surface area defects located in lateral breast or superior medial quadrant. Main advantages include muscle-sparing approach, preserved shoulder strength, reduced post-operative pain and seroma, and maintenance of the posterior axillary fold [66,67,68,69].
Regional Flaps	Latissimus Dorsi (LD) flap	Vascular supply is via the thoracodorsal artery. Main advantages include reliable perfusion and relative ease of flap harvest. Disadvantages include donor site morbidity, long back scar, high seroma rates, and increased postoperative time due to need for position changes during flap harvest and inset [72,73].
	Thoracoepigastric flap	Vascular supply is through perforators from the superior epigastric artery. Suitable for defects located in inferior half of breast, both lateral and medial. Main advantages include good volume match and low donor site morbidity in properly selected patients (those with adequate subcutaneous tissue and skin in the upper abdomen with no prior scarring in the ipsilateral upper abdomen, as their pedicle may be compromised) [61,74,75].
	Omental flap	Vascular supply is through the left or right gastroepiploic arteries. Suitable for medial breast defects, especially when >20% of breast tissue is excised. Main advantages include the omentum’s unique regenerative, radiolucent, and infection-resistant properties. Can be laparoscopically harvested, offering low donor site morbidity. Disadvantages include volume insufficiency for large defects and risk of fat necrosis [76,77]
Free tissue transfers	Deep Inferior Epigastric Perforator (DIEP) flap	Vascular supply is through perforators of the deep inferior epigastric artery. Suitable for large-volume defects in patients with adequate lower abdominal tissue. Main advantages include robust volume restoration and preservation of abdominal muscle compared to TRAM flaps. Disadvantages include need for microsurgical expertise, long operative time and preclusion of future abdominally-based reconstruction, which is important for patients who may later require mastectomy after positive margins [78].
	Superficial Inferior Epigastric Artery (SIEA) flap	Vascular supply is through the superficial inferior epigastric artery. Suitable for similar indications as the DIEP flap but only in patients with favorable superficial vascular anatomy. Main advantages include avoidance of intramuscular dissection. Disadvantages include unreliable vascular anatomy in many patients and same limitation as DIEP—precludes future abdominally-based reconstructions [79].
	Medial Circumflex Femoral Artery Perforator flap	Vascular supply is through perforators of the medial circumflex femoral artery. Suitable for moderate-volume defects, particularly in central breast locations where pedicled flaps are less feasible. Main advantages include reliable vascularity and discreet donor site. Disadvantages include smaller volume yield and shorter pedicle lengths compared to other free flaps [80].
	Superficially based low-abdominal mini (SLAM) flap	Vascular supply is through either the superficial inferior epigastric artery or the superficial circumflex iliac artery, depending on dominance. Suitable for small-breasted, thin patients with 20–50% volume excision desiring volume preservation. Main advantages include preservation of abdominal tissue for future reconstruction need. Disadvantages include limited flap volume [81].
**Hybrid Technique**		
Biplanar oncoplastic approach		Combines glandular reshaping with small-volume subpectoral implants. Suitable for selected patients with moderate sized defects desiring volume preservation. Main advantages include improved breast projection and symmetry when glandular reshaping alone is insufficient. Disadvantages include potential implant-related complications [57].

## Abbreviations

The following abbreviations are used in this manuscript:

OBCS	Oncoplastic Breast-Conserving Surgery
BCS	Breast-Conserving Surgery
BCT	Breast-Conserving Therapy
ASBS	American Society of Breast Surgeons
CPT	Current Procedural Terminology
NAC	Nipple-Areolar Complex
IMAP	Internal Mammary Artery Perforator
AICAP	Anterior Intercostal Artery Perforator
LICAP	Lateral Intercostal Artery Perforator
LTAP	Lateral Thoracic Artery Perforator
TDAP	Thoracodorsal Artery Perforator
LD	Latissimus Dorsi
DIEP	Deep Inferior Epigastric Perforator
SIEA	Superficial Inferior Epigastric Artery
SLAM	Superficially-based Low-Abdominal Mini
BMI	Body Mass Index
